# The top 100 most cited articles in helical tomotherapy: a scoping review

**DOI:** 10.3389/fonc.2023.1274290

**Published:** 2023-10-17

**Authors:** Yadong Zhang, Liyi Rong, Zhiqiang Wang, Hongfu Zhao

**Affiliations:** Department of Radiation Oncology, China-Japan Union Hospital of Jilin University, Changchun, China

**Keywords:** most cited articles, scoping review, helical tomotherapy, research trends, network visualization

## Abstract

**Objective:**

The purpose of this scoping review was to explore the top 100 most cited articles in helical tomotherapy (HT) through bibliometric analysis and visualization tools, help researchers comprehensively understand the research hotspots of HT, and provide clear and intuitive network visualization.

**Methods:**

The Web of Science Core Collection and the search strategy of “Title (TI)=(tomotherapy)” were used to search for articles related to HT as of 27 May 2023. The top 100 most cited articles were obtained by sorting “citations: highest first”. From these top 100 most cited articles, the following information was extracted: journals, years and months, countries, authors, types of tumor treated, and topics. The VOSviewer software was introduced for visualizing all the articles related to HT.

**Results:**

The top 100 most cited articles in HT were published between 1999 and 2019. The citation counts of these articles ranges from 326 to 45, with a total of 8,422 citations at the time of searching. The index of citations per year (CPY) ranges from 22.32 to 2.45. These articles originated from 17 countries, with most publications from the United States (n=50), followed by Canada (n=12), Italy (n=10), Germany (n=7) and Belgium (n=5). The International Journal of Radiation Oncology, Biology, Physics published the highest number of articles (n=31), followed by Radiotherapy and Oncology (n=20), Medical Physics (n=13) and Strahlentherapie und Onkologie (n=12). In terms of specific tumor types, head and neck cancer (n=15) is the most common disease, followed by cancers with complex target structures (n=14), breast cancer (n=12), prostate cancer (n=10) and lung cancer (n=8). The most common research topics also include dosimetric comparison (n = 44), quality assurance (n = 12) and Megavoltage CT (n = 8).

**Conclusion:**

This scoping review provides a comprehensive list of the 100 most cited articles in HT. This analysis offers valuable insights into the current research directions of HT that can be utilized by researchers, clinicians, and policy-makers.

## Introduction

Helical tomotherapy (HT) is an advanced rotational technique that, in the manner of a computed tomography (CT) scanner, uses a fan-beam to deliver intensity-modulated radiation therapy (IMRT) treatments (1, 2). The combination of the integrated megavoltage (MV) CT imaging system, the binary pneumatic multi-leaf collimator with fast leaf open times (about 20ms), and the extended treatment field size along the patient’s axis offers numerous advantages, particularly for treating cancers with complex and large targets (CCLT) accurately (3–5). With the recent introduction of the kilovoltage (kV) CT imaging, HT can provide intra-fractional real-time tumor tracking by means of the Synchrony technology adapted from Cyberknife (6). Meanwhile, HT has the ability of inter-fractional adaptive radiotherapy, which means that during the process of radiotherapy, new imaging sets are considered to accommodate changes in anatomical structures and tumor shrinkage, resulting in the generation of new treatment plan (7). As a result, HT opens up the opportunity for dose escalation, a method that increases radiation dosage to effectively target cancer cells while minimizing the damage to healthy tissues (8–12). By enabling more precise radiation delivery and minimizing unnecessary exposure to healthy tissues, HT allows medical professionals to optimize treatment plans and enhance the effectiveness of radiotherapy.

Several studies have previously focused on discussing the current state and progress of HT in certain specific fields (13, 14). However, there were no bibliometric analyses of literature in the field of HT using VOSviewer (version 1.6.13, Leiden University, Leiden, Netherlands) software. As a bibliometric analysis tool, the VOSviewer has the ability to quickly process a large number of references and has visual output of analysis results that are not available in “classical” bibliometric analysis. As far as we are aware, this study represents one of the early attempts to utilize VOSviewer visualization technology in order to gain a better understanding of the global research trends and prominent areas of research in HT.

The purpose of this scoping review was to explore the top 100 most cited articles in HT through bibliometric analysis and visualization tools, help researchers comprehensively understand the research hotspots of HT, and provide clear and intuitive network visualization.

## Methods

### Search strategy

This study did not require ethics approval as it solely relied on data from publicly available publications. Since our study is a scoping review, it was reported according to the PRISMA guidelines, see **Table S1**. The Web of Science Core Collection (WOSCC) database was used to search for relevant studies in HT. The search strategy used to identify these studies was “Title (TI)=(tomotherapy)”, and the search was restricted to English language. The search covered a time period ranging from 1900 when the database was established to May 27, 2023. WOSCC provides a total citation count for each articles searched and a ranking of citation counts from high to low. The reasons for choosing the WOSCC database to obtain the top 100 most cited articles have been explained in previous study (15). That is, there are several literature databases, such as WOSCC, Google Scholar, and Scopus, which provide citation count data queries. Journals and articles covered by different databases are different. Thus, the number of citations to the same article in different databases is different. Google Scholar is a freely accessible web search engine that uses a web crawler, or web robot, to identify files for inclusion in the search results, resulting in inconsistent accuracy in the results it provides. In terms of quality and quantity, such as coverage, citations, search and special features, etc., the Scopus and the WOSCC database are equally competitive, with each having its own advantages. However, due to WOSCC’s longer establishment history and being the only literature database before the establishment of Scopus, WOSCC is still more popular worldwide at this stage (16).

### Data extraction and analysis

Due to the fact that studies published earlier tend to accumulate more citations, the researchers adjusted for the year and month of publication by calculating a citations per year (CPY) index. This index was obtained by dividing the total citations by the number of years of publication up until May 2023 for each article. The top 100 most cited articles were normalized to CPY. From the top 100 most cited articles, the following information was extracted: journals, years and months, countries, authors, types of tumor treated, and topics. The classification of types of tumor and topics was conducted by two independent authors. In cases where discrepancies arose, a third author was consulted to resolve them. If clinical cases were involved in an article, it would be classified into the relevant type of tumor, such as head and neck cancer, breast cancer, etc. If there was dosimetric comparison in an article, it would be classified as a dosimetric comparison. Quality assurance and MVCT are similar. If several types of tumor or topics were covered in one study, count for each one. Through the discussions, we can delve into the trends, patterns, and advancements related to these specific aspects. This will enable us to comprehend the significance, impact, and scope of research in the field of HT, thereby gaining a comprehensive understanding of the research hotspots and trends in HT. Additionally, through these discussions, we can identify potential knowledge gaps, determine areas for further research, and potentially contribute to the field.

The top 100 most cited articles may not cover current research, as citations require time. In order to comprehensively analyze HT related research that includes current research, the VOSviewer software was introduced for network visualization, including journals, countries, authors, and maps, to encompass all articles related to HT.

## Results

In total, 3,099 articles from 1999 to 2023 were searched from database. The top 100 most cited articles in HT were included in the scoping review (see **Figure S1**) published between 1999 and 2019, and the citations ranged from 326 to 45, which collectively had been cited 8,422 times at the time of searching. The index of citations per year (CPY) ranges from 22.32 to 2.45, see **Table S2**. The International Journal of Radiation Oncology, Biology, Physics published the highest number of articles (n=31), followed by Radiotherapy and Oncology (n=20), Medical Physics (n=13) and Strahlentherapie und Onkologie (n=12), see **Table 1**.

**Table 1:** Journals, in which the 100 most cited articles of helical tomotherapy were published.

Journal	No. of articles	2021 IF	Category and 2021 JCI quartile
International journal of radiation oncology biology physics	31	8.013	Oncology Q1
Radiotherapy and oncology	20	6.901	Oncology Q1
Medical physics	13	4.506	RNMM Q1
Physics in medicine and biology	12	4.174	Engineering, biomedical Q2
Strahlentherapie und onkologie	4	4.033	Oncology Q2
Technology in cancer research & treatment	3	2.876	Oncology Q4
British journal of radiology	3	3.629	RNMMI Q2
Seminars in radiation oncology	2	5.421	Oncology Q2
Medical dosimetry	2	1.531	Oncology Q4
Radiation oncology	2	4.309	Oncology Q2
Clinical oncology	2	4.925	Oncology Q2
Journal of applied clinical medical physics	1	2.243	RNMMI Q3
Acta oncologica	1	4.311	Oncology Q2
American journal of clinical oncology-cancer clinical trials	1	2.787	Oncology Q4
Neurosurgery	1	5.315	Clinical neurology Q1
Physica medica-european journal of medical physics	1	3.119	RNMMI Q3
Bone marrow transplantation	1	5.174	Hematology Q1

IF, Impact Factor; JCI, Journal citation Indicator; Q, Quartile; RNMMI, Radiology, nuclear medicine and medical imaging.

**Figure 1:**
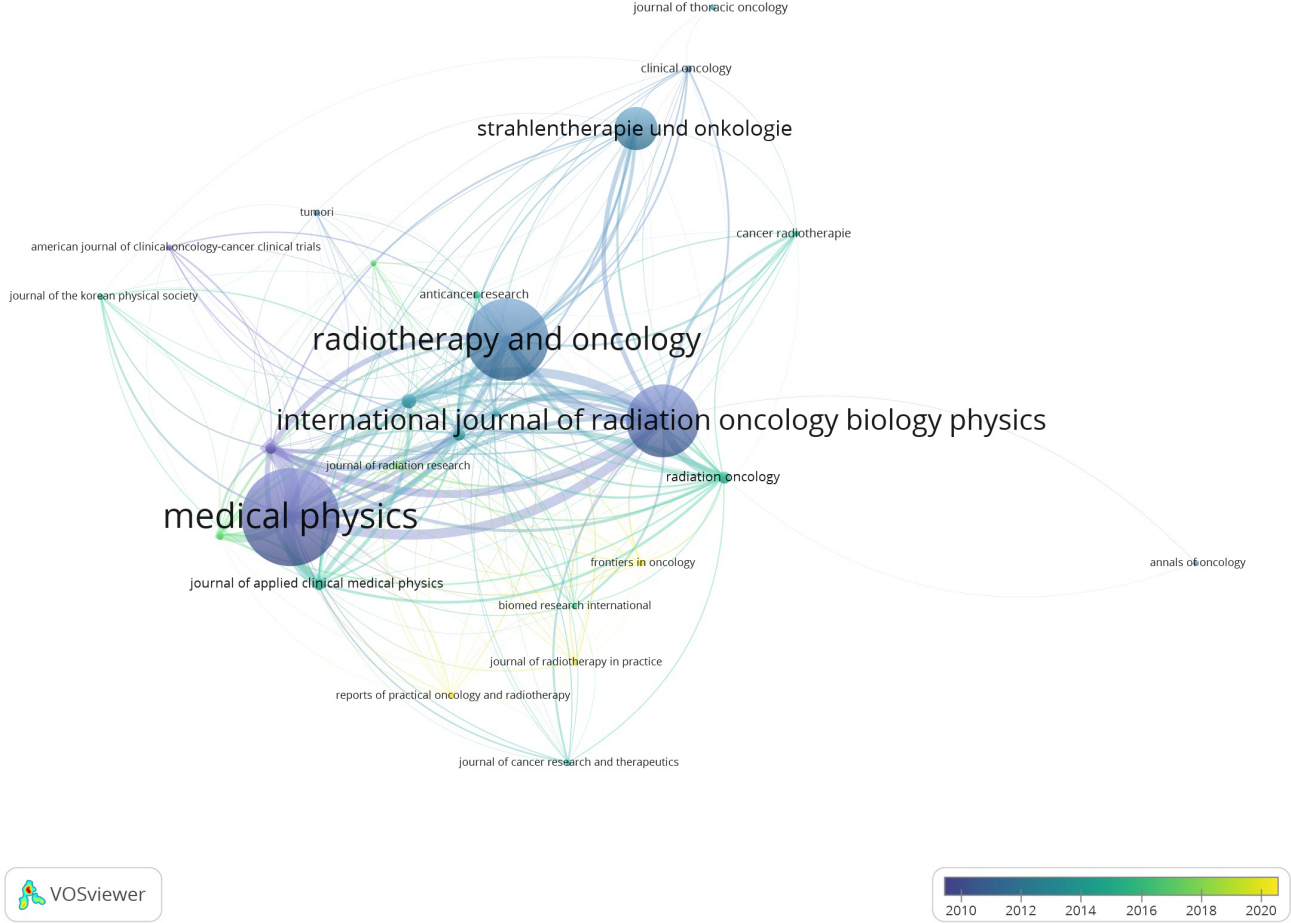
The network visualization of sources of citation according to the average publication year.

Among all 3,099 articles, Medical Physics has published the most articles (n=709), followed by Radiotherapy and Oncology (n=587), International Journal of Radiation Oncology Biology Physics (n=504) and strahlentherapie und onkologie (n=272), see **Figure 1**. The links between the journals represent citations, where a paper was published in one journal and cited by another journal. In the network visualization, the circle size represents the number of occurrences. The larger the circle is, the greater the occurrence. The width of the curved line represents the link strength. The wider the line is, the more links there are. The distance between two occurrences approximately indicates the relatedness of the nodes. The color of a journal in **Figures 1** represents the average year of publication for all articles related to the journal, rather than the first or last publication year. The color legend was shown in the bottom right corner of the figures. Purple represents the earliest year, while yellow represents the latest year.

**Figure 2:**
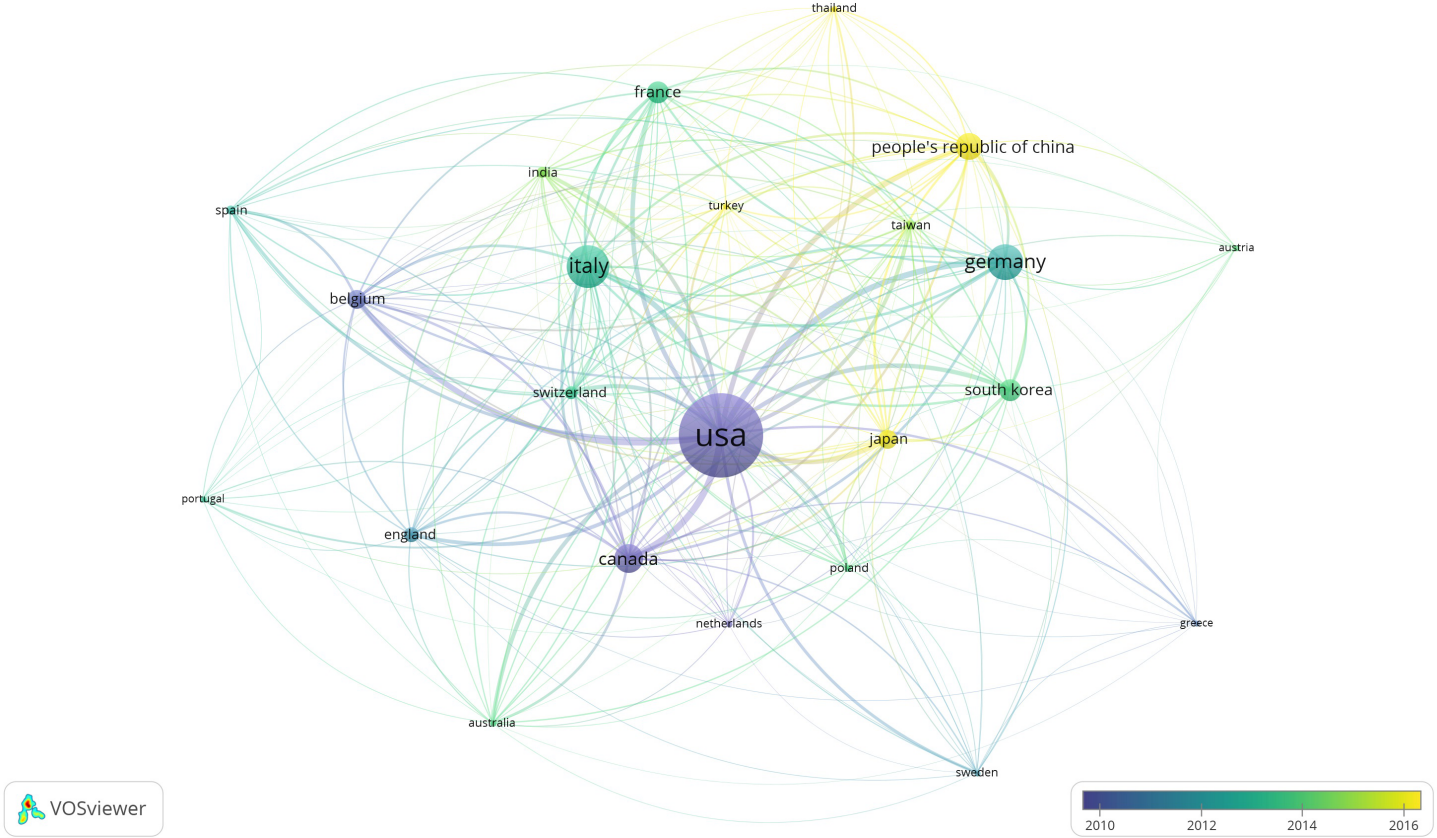
The network visualization of countries of co-authorship according to the average publication year.

The top 100 most cited articles originated from 17 countries, with most publications from the United States (n=50), followed by Canada (n=12), Italy (n=10), Germany (n=7) and Belgium (n=5), see **Table 2**. The network visualization of countries of co-authorship for all articles related to HT according to the average publication year was shown in **Figure 2**.

**Table 2:** Countries of the 100 most cited articles in the field of helical tomotherapy.

Country or origin	Number of articles ^*^
The United States	50
Canada	12
Italy	10
Germany	9
Belgium	5
England	3
Taiwan, China	3
India	2
Korea	2
Australia	1
Egypt	1
France	1
People’s Republic of China	1
Slovenia	1
Spain	1
Switzerland	1
Turkey	1

^*^Several articles were from multiple countries (count for each country).

Twelve first authors were credited with having no fewer than two articles among the top 100 most cited articles. The list of first author, corresponding author, and co-author with the largest number of the top 100 most cited articles was shown in **Table 3**. The network visualization of authors of co-authorship for all articles related to HT according to the average publication year was shown in **Figure S2**.

**Table 3:** Number of authorships of the top 100 most frequently cited articles in the field of helical tomotherapy.

Description	Author name (number of articles)
Most frequent first author	Fiorino, C (3); Kapatoes, JM (3); Penagaricano, JA (3); Sterzing, F (3); Balog, J (2); Bauman, G (2); Fenwick, JD (2); Oliver, M (2); Ruchala, KJ (2); Schubert, LK (2); Sheng, K (2); Welsh, JS (2)
Most frequent corresponding author	Fiorino, C (3); Kapatoes, JM (3); Penagaricano, J (3); Tome, WA (3); Balog, J (2); Bauman, G (2); De Ridder, M (2); Fenwick, JD (2); Kron, T (2); Mahan, SL (2); Oliver, M (2); Ruchala, KJ (2); Schubert, LK (2); Sheng, K (2); Shepard, DM (2); Sterzing, F (2); Welsh, JS (2)
Most frequent co-author (total)	Mackie, TR (22); Olivera, GH (17); Mehta, M (12); Kapatoes, JM (10); Tome, W (10); Broggi, S (8); Calandrino, R (8); Fiorino, C (8); Reckwerdt, P (8); Ruchala, K (8); Di Muzio, N (7); Fazio, F (7); Schubert, K (7); Bauman, G (5); Dell’Oca, I (5); Jaradat, H (5); Paliwal, B (5)

Head and neck cancer (n=15) is the most common diseases, followed by CCLT (n=14), breast cancer (n=12), prostate cancer (n=10) and lung cancer (n=8). Dosimetric comparison (n = 44) is the most common topic, followed by quality assurance (QA) (n = 12), MVCT (n = 8) and clinical research (n = 4), see **Table 4**. The network visualization of map based on title and abstract for all articles related to HT according to the average publication year was shown in **Figure S3**.

**Table 4:** Type of tumor treated, and topics of the 100 most cited articles on helical tomotherapy.

Tumor or topic	Number of articles (rank in Table S2)
Type of tumor	
head and neck cancer	15 (6, 11, 14, 19, 20, 27, 36, 39, 41, 55, 64, 72, 90, 91, 96)
cancers with complex and large targets^*^	14 (3, 9, 16, 17, 35, 54, 57, 62, 74, 78, 88, 95, 98, 99)
breast cancer	12 (13, 18, 26, 32, 44, 47, 56, 60, 65, 82, 87, 94)
prostate cancer	10 (2, 6, 30, 33, 37, 42, 53, 61, 70, 85)
lung cancer	8 (6, 29, 50, 51, 58, 76, 79, 80)
Topic	
dosimetric comparison	44 (2, 3, 6, 13, 14, 16, 18, 19, 20, 26, 27, 30, 31, 32, 33, 35, 36, 39, 40, 41, 42, 44, 47, 51, 52, 54, 55, 56, 57, 60, 62, 64, 65, 66, 67, 72, 74, 78, 80, 82, 84, 87, 94, 96)
quality assurance	12 (7, 10, 24, 34, 38, 46, 68, 69, 73, 92, 93, 97)
megavoltage CT	8 (4, 8, 22, 28, 45, 49, 53, 61)
clinical research	4 (37, 58, 70, 71)

^*^craniospinal axis irradiation, hippocampal-sparing whole-brain radiotherapy, malignant pleural mesothelioma, total body irradiation, total bone marrow, total lymphatic, whole abdominal irradiation.

## Discussion

### VOSviewer

This study identifies the journals, countries, and authors that have made outstanding contributions in the field of HT. Overall, Medical Physics have made significant contributions in the early years of HT applications, including the concept of HT, equipment development and validation, feasibility studies for CCLT, and QA, as their average publication year is significantly earlier. Compared to the frequency of journals in the top 100 most cited articles, it is clear that “Medical Physics” has an advantage in terms of overall quantity, demonstrating its influence in the field. This also highlights that HT is an advanced technology that requires a significant amount of QA program as its foundation. International Journal of Radiation Oncology Biology Physics and Radiotherapy and Oncology, as top journals in the field of radiation therapy, have made impressive contributions in publishing HT research.

Regardless of the total articles or the top 100 most cited articles, the United States is the most influential country and has made more and earlier researches in this field. Canada, Italy, and Germany also have a considerable total number of articles, as well as the top 100 most cited articles. There was close cooperation between countries, especially, the United States has the highest value of centrality, which may be related to the fact that the United States is the birthplace of HT, and the higher available of the HT.

Each of the top 8 authors (Fiorino C, Calandrino R, Di Muzio N, Broggi S, Papanikolaou N, Olivera G, Chen Q and Chen Y) in this field has made significant contributions to the literature, with each author having published a minimum of 40 articles. Among the “prolific authors”, it is worth noting that Fiorino C, Calandrino R, Di Muzio N and Broggi S work in the same clinic that firstly commissioned an HT machine in Italy. Their extensive work demonstrates their dedication and expertise in the field. Their contributions have not only enriched the academic literature but have also had an impact on the field as a whole.

### Head and neck cancer

Head and neck cancer have attracted significant attention from oncologists and medical physicists due to their complex anatomical structure. Nasopharyngeal carcinoma (NPC) stands as a prominent representative within this category. Among the top 100 most cited articles in HT, 15 publications specifically addressed patients with head and neck tumors, with 4 of them focused exclusively on NPC (9, 17–19). Widesott et al. conducted a comparison between two irradiation techniques, intensity-modulated proton therapy (IMPT) and HT in treating nasopharynx cancer using a simultaneous integrated boost (SIB) approach (17). The results showed that both IMPT and HT demonstrated excellent target coverage, homogeneity within the PTVs, and spared the organs at risk effectively. A study conducted by Lu et al. aimed to compare the effectiveness of three different radiation therapy techniques, namely volumetric modulated arc therapy (VMAT), HT, and IMRT, for treating NPC patients (18). The researchers found that VMAT treatment provides better sparing of OARs, improved homogeneity and conformity compared to IMRT. Furthermore, it was observed that VMAT treatment provided shorter delivery times compared to HT. Current research can focus on shortening delivery time to better manage time and improve the efficiency of medical institutions. Studies from Fiorino et al. demonstrated that HT improved the homogeneity of dose distribution within the PTV and increased the coverage of PTV_54Gy_ and PTV_64.5Gy_ compared to IMRT (9). Furthermore, the study also found significant improvements in sparing most OARs with HT compared to IMRT. The research conducted by Lee et al. has also yielded similar findings, which suggest that HT may be a more effective treatment option for NPC (19).

### CCLT: cranial spinal irradiation

Cranial spinal irradiation is mainly indicated for patients diagnosed with CNS tumors, such as medulloblastoma (20, 21), germinoma (22), etc., which is a typical case with complex and large targets and is one of the typical indications for HT. In comparison to other techniques, HT provides superior target coverage, ensuring that the radiation is delivered uniformly and consistently throughout the affected area. This precision is critical for ensuring effective treatment outcomes and minimizing the risks of under or over-treatment. One notable aspect of HT is its ability to spare critical organs at risk (OARs), such as the thyroid, parotids, cochlea, eyes, heart, and esophagus (8, 23). By precisely shaping and directing the radiation beams, HT minimizes the radiation dose absorbed to OARs, reducing the potential for collateral damage and associated toxicities. This organ-sparing feature greatly contributes to the safety and efficacy of HT for cranio-spinal axis treatment.

However, it is worth noting that one drawback of HT is the longer beam-on-time (BOT) compared to other techniques. This is primarily due to the complexity of the rotational IMRT delivery and the need for multiple treatment beams. Study from Turcas et al. have reported that the BOT for HT was significantly longer than that of other techniques, with a median BOT of 11 minutes for HT, 5.49 minutes for VMAT, and 1.46 minutes for three dimensional conformal radiation therapy (3D-CRT) (13). While this extended treatment time may be a consideration, the superior clinical outcomes and reduced toxicities associated with HT for cranio-spinal axis treatment suggest that the benefits outweigh this potential drawback (20–22).

### CCLT: total body irradiation and total marrow irradiation

In 2005, Hui et al. conducted experiments by varying the pitch, field width, and modulation factor, verifying the feasibility of total body irradiation (TBI) and total marrow irradiation (TMI) using HT (24). They achieved a uniform dose distribution while maintaining treatment times comparable to conventional TBI (15-30 minutes). To evaluate the accuracy of dose delivery, thermoluminescent detectors (TLDs) were placed inside a Rando phantom at seven anatomical sites, including the lungs. The results of their simulated TBI treatment demonstrated a homogeneous dose coverage ( ± 10%) across the entire body, with significant reduction (35%-70%) in doses to sensitive organs compared to the target dose. In the TMI study, the dose was conformed specifically to the bone marrow. It established the feasibility of TBI or TMI using HT and provided important planning parameters and reference for dose constraints for OARs. Peñagarícano et al. validated the feasibility of TBI using HT for the treatment of four patients with acute myeloid leukemia (AML), verified the accuracy of the delivered dose and analyzed any toxicity or side effect through an evaluation on the treatment planning process, delivery of the treatment (25). To verify the accuracy of the delivered dose, the authors reconstructed the dose by contouring specific areas of interest on the daily pretreatment MVCT scans for each fraction. A deformable registration model was used to sum up the doses from all individual fractions. By comparing the planned doses with the delivered doses, they found that there were small differences, with the average of 2.7%. This indicated that the treatment planning process and delivery of the treatment with HT were precise and accurate. In terms of toxicity, the TBI treatment was generally well-tolerated, with only mild side effects observed. Outcomes for the patients suggested that the HT-supported TBI treatment was effective in controlling the disease, although further research was needed to fully understand its impact on patient survival and disease progression. These findings supported the potential of HT as a viable treatment option for TBI in patients with AML. Gruen et al.’s study enrolled a total of 10 patients with acute lymphoblastic leukemia or AML who received TBI with HT (12). The results showed improved dose distribution and homogeneity, as well as selective dose reduction to organs at risk. No grade 3 or 4 side effects was observed.

### CCLT: malignant pleural mesothelioma

Sterzing et al. evaluated the potential of HT in the adjuvant treatment of malignant pleural mesothelioma and compare target homogeneity, conformity and normal tissue dose with step-and-shoot IMRT (10). Both HT and step-and-shoot IMRT achieved excellent dose distributions while minimizing damage to organs at risk. However, HT demonstrated significant improvements in target coverage and homogeneity compared to step-and-shoot IMRT. Additionally, it was found that the mean dose to the contralateral lung could be reduced by more than 5 Gy, indicating better normal tissue sparing.

### CCLT: whole abdominal irradiation

Rochet et al. demonstrated that HT for delivering whole abdominal irradiation (WAI) in patients with advanced ovarian cancer. It provided excellent coverage of the PTV while effectively sparing the liver, kidneys, and bone marrow from excessive radiation exposure (11).

### Breast cancer

Schubert et al.’s study demonstrated that HT resulted in lowest heart and ipsilateral lung max doses, but had higher mean doses compared to 3D-CRT and IMRT (26). Moon et al. compared the dosimetry of four different external beam accelerated partial breast irradiation (APBI) plans: 3D-CRT, IMRT, HT, and proton beam therapy (27). All four APBI techniques showed acceptable coverage of the PTV. However, effective non-PTV breast sparing was achieved at the cost of considerable dose exposure to the lung and heart in HT. This was consistent with the research results of Caudrelier et al. (28).

### Prostate cancer

Prostate cancer is one of the most common malignant diseases in men. Based on CT datasets of nine prostate cancer patients, Wolff et al. evaluated the plan quality and delivery efficiency of several treatment techniques, such as 3D-CRT, IMRT, VMAT, and HT (29). This article, ranked 2nd in terms of total citations with a total of 279, and ranked 1st in terms of CPY with a score of 22.3, had important value in the dosimetry comparative study of prostate cancer. This study suggests that all other approaches yield treatment plans of improved quality when compared to 3D-CRT, with HT providing best OAR sparing and VMAT being the most efficient treatment option. This conclusion was supported by other highly cited articles (30–32).

### Lung cancer

Lung cancer is a major source of patients for treatment with HT, and due to the characteristics of HT treatment, the interplay effect is one of the factors that must be considered (33–35). Although several studies have shown that the dose uniformity perturbation was not significant at the typical breathing frequency and amplitude in HT delivery (36, 37). But radiation oncologists and medical physics should always pay attention to this effect.

### QA of the integrated components

Compared to C-arm accelerators, considering the unique design of HT, it is crucial to develop a set of QA recommendations specifically tailored to HT. Among the top 100 most cited articles in the field of HT, 12 of them specifically focus on QA, see **Table 4**. One particularly notable article, ranked 7th in terms of total citations with a total of 167, and ranked 5th in terms of CPY with a score of 14.3, is the task group 148 report for the QA of HT (38). This article extensively discussed various QA techniques, recommended frequencies for QA assessments, and tolerances. It provided practicing clinical medical physicists with valuable insights into the technology and knowledge required to establish an independent and comprehensive QA program specifically for HT. The modified MapCHECK with less than ± 2% sensitivity change with incident angle can meet the validation of the treatment plan for HT. The gamma pass rates of 3mm/3% and 2mm/2% of HT treatment plans for ten patients, including prostate cancer, head and neck cancer, esophageal cancer, cervical cancer, etc., were 97.6% to 100% and 91.8% to 98.2%, respectively (39).

MVCT is a major feature of HT, which offer verification of the patient position prior to and potentially during radiation therapy. Although MVCT images do not have the same performance characteristics as advanced diagnostic CT scanners when one objectively examines noise and low-contrast resolution, these images are useful not only for verifying the patient’s position at the time of therapy, but they are also sufficient for delineating many anatomic structures (4, 40–43). The combination of MVCT image sets and a measured database of detector signal provides a fast dose reconstruction for online delivery verification. The performance evaluation of this approach was divided into two steps. The first step was to verify the energy fluence. Energy fluence verification was performed using the pulse-by-pulse approach for the measured and database dose matrices. The second step was to conduct dose verification. Doses were compared between the dose calculated using previously verified energy fluence with the dose reconstructed using database-based approach. Simulating prostate delivery on an inhomogeneous abdominal phantom and nasopharyngeal delivery on a dog cadaver revealed that this approach creates an opportunity for real-time delivery verification and dose reconstruction (44).

### Significance

This bibliometric analysis has identified and listed the top 100 most cited articles in HT. These articles stand out for their outstanding contributions to the concept of HT, equipment development and validation, feasibility studies for CCLT, and QA. Whilst this article looks only at a few features of the included articles, it is a neat overview of the research and most cited publications in HT. For physicists and physicians involved in HT, particularly those who are relatively new to the field with less than 5 years of experience, these articles can prove to be invaluable resources. They provide comprehensive insights, treatment plan parameters, dose constraints to OARs, and confidence in dose delivery that can enhance their understanding and skills in HT. By exploring these articles, these professionals can gain in-depth knowledge about the latest advancements, effective treatment techniques, and best practices for optimizing patient outcomes.

### Limitations

This study has some limitations that need to be considered. First, using simple search text and a single publication database may miss potential studies that have reported on the topic of HT. Therefore, there is a chance that valuable insights and findings from these studies might have been excluded from the analysis. Moreover, the objective evaluation of the number of citations as a measure of the impact and quality of the studies may also be compromised in a few ways. Journal self-citations and author self-citations could inflate the number of citations for certain articles. This could lead to an overestimation of the importance or influence of these studies in the research community. Furthermore, it is worth noting that relying solely on citation counts may result in some articles with low citation counts and citation per year (CPY) to be overlooked in this study. These articles may still contain valuable insights or unique research contributions but have not received significant attention or recognition in the academic community.

## Conclusion

To our knowledge, this report represents the first bibliometric analysis of the top 100 most cited articles in HT, revealing its historical developments and significant advancements. These articles serve as valuable resources for researchers, clinicians, and policy-makers seeking to deepen their understanding of this field. The report not only presents network visualizations, including journals, countries, authors, and maps but also offers valuable insights into past and current research in HT and may be used to shape current research directions, based on perceived research gaps. HT exhibits unique advantages in the treatment of CCLT, although it is worth noting that longer delivery time could be seen as a potential drawback. Current research in this field should place emphasis on reducing the delivery time of HT while maintaining its dosimetric advantages. Moreover, it is worth exploring how to translate these advantages into improved clinical outcomes or reduced side effects.

## Data availability statement

The original contributions presented in the study are included in the article/**Supplementary Material**, further inquiries can be directed to the corresponding author.

## Author contributions

YZ: Conceptualization, Data curation, Visualization, Methodology, Writing – original draft. LR: Conceptualization, Data curation, Visualization, Methodology, Writing – review & editing. ZW: Visualization, Writing – original draft, Data curation. HZ: Conceptualization, Methodology, Writing – review & editing.
