# Supplementary figures and images for: The top 100 most cited articles in helical tomotherapy: a scoping review
Source: Front Oncol. 2023 Oct 17;13:1274290. doi: 10.3389/fonc.2023.1274290 (PMC10616822; doi:10.3389/fonc.2023.1274290)

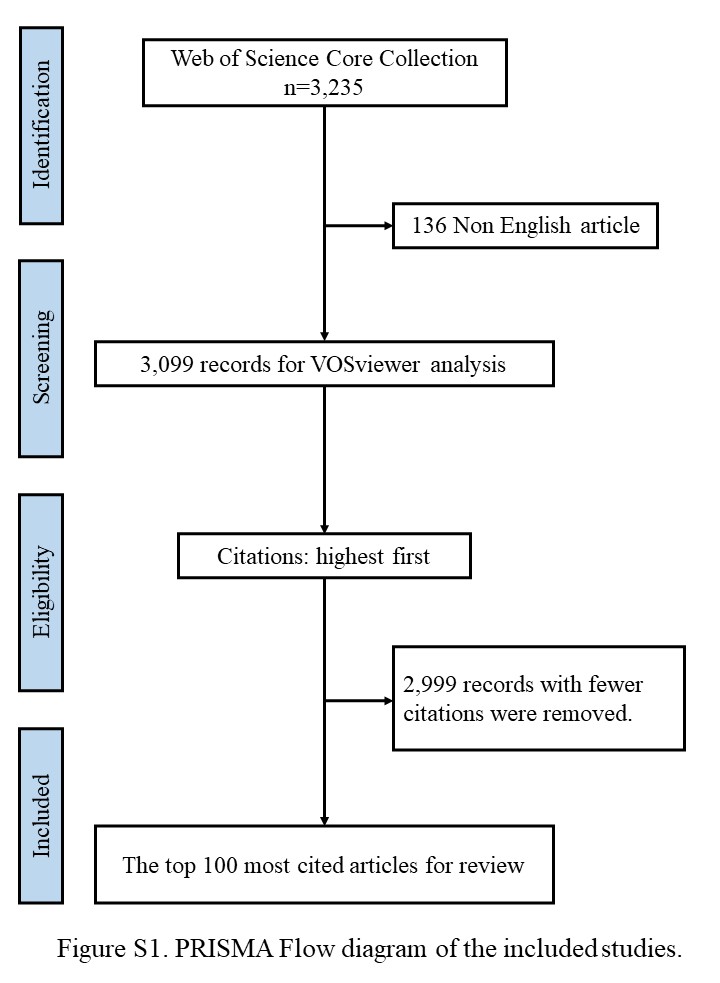

Supplement: Supplementary file 1 [file Image_1.jpeg]

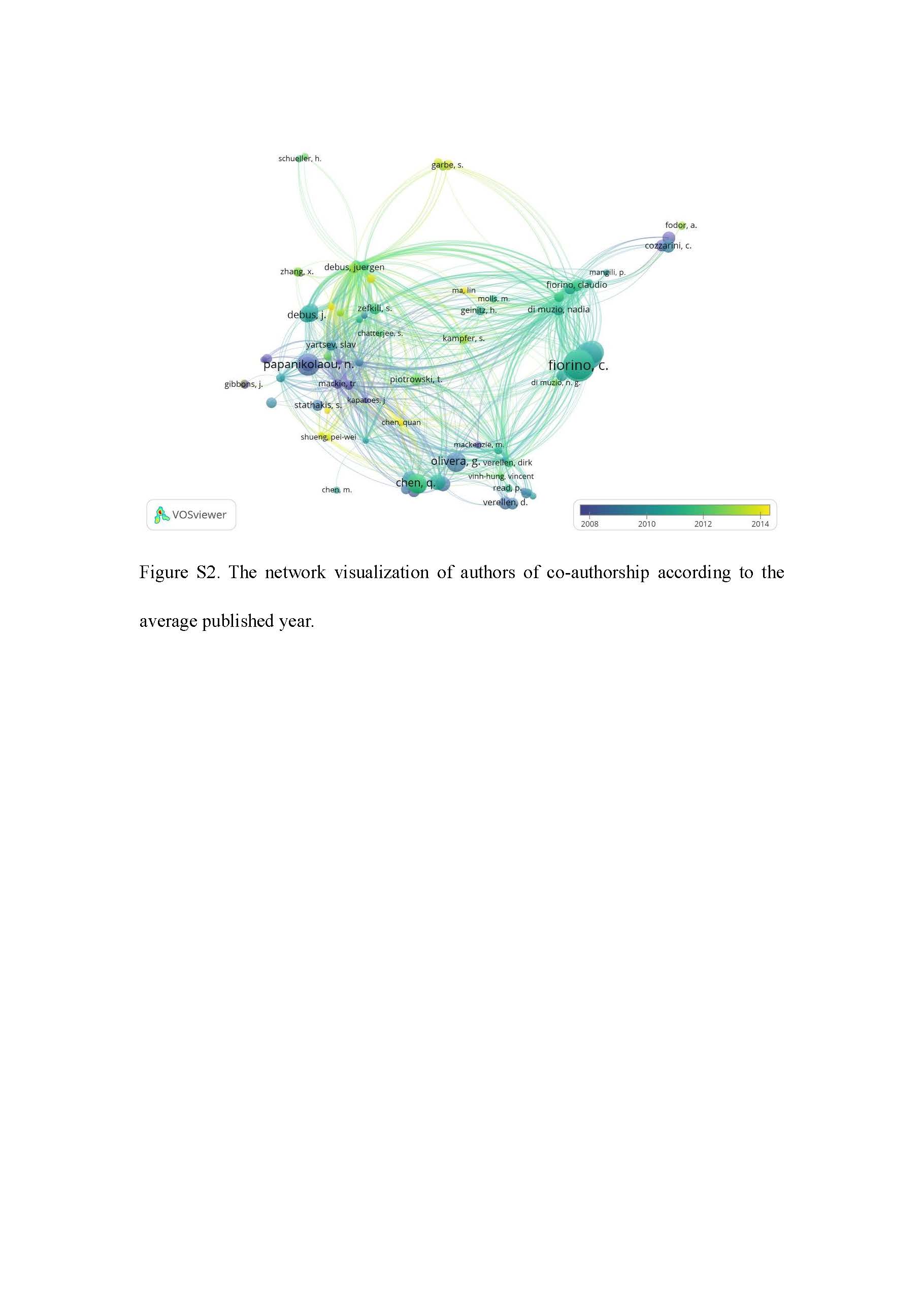

Supplement: Supplementary file 2 [file Image_2.jpeg]

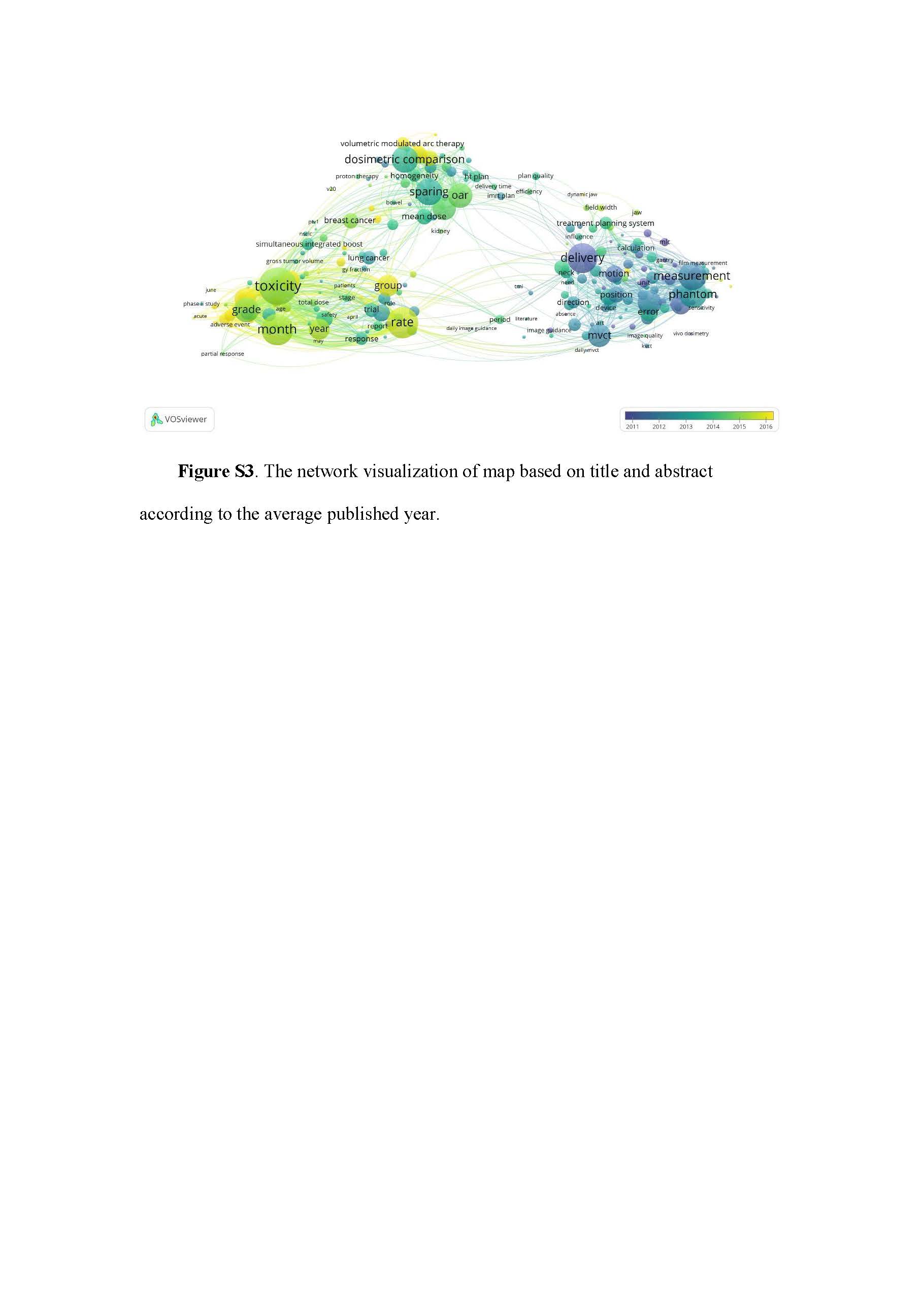

Supplement: Supplementary file 3 [file Image_3.jpeg]
