# Supplementary material for: The top 100 most cited articles in helical tomotherapy: a scoping review
Source: Front Oncol. 2023 Oct 17;13:1274290. doi: 10.3389/fonc.2023.1274290 (PMC10616822; doi:10.3389/fonc.2023.1274290)
Supplement: Supplementary file 5 [file Table_2.docx]

Table S2

One hundred most cited articles on Tomotherapy, ranked in order of number of citations received

| Rank | Year | Journal | Title | TC | CPY (rank) |
| --- | --- | --- | --- | --- | --- |
| 1[[1](#_ENREF_1)] | 1999 | SRO | Tomotherapy | 326 | 14.0 (6) |
| 2[[2](#_ENREF_2)] | 2009 | RAO | Volumetric modulated arc therapy (VMAT) vs. serial tomotherapy, step-and-shoot IMRT and 3D-conformal RT for treatment of prostate cancer | 279 | 22.3 (1) |
| 3[[3](#_ENREF_3)] | 2010 | IJROBP | Hippocampal-sparing whole-brain radiotherapy: a "how-to" technique using helical tomotherapy and linear accelerator-based intensity-modulated radiotherapy | 231 | 20.1 (2) |
| 4[[4](#_ENREF_4)] | 1999 | PMB | Megavoltage CT on a tomotherapy system | 204 | 9.0 (12) |
| 5[[5](#_ENREF_5)] | 2006 | PMB | History of tomotherapy | 203 | 12.8 (7) |
| 6[[6](#_ENREF_6)] | 2010 | MP | Comparison of Elekta VMAT with helical tomotherapy and fixed field IMRT: Plan quality, delivery efficiency and accuracy | 177 | 14.5 (4) |
| 7[[7](#_ENREF_7)] | 2010 | MP | QA for helical tomotherapy: Report of the AAPM Task Group 148 | 167 | 14.3 (5) |
| 8[[8](#_ENREF_8)] | 2005 | MP | Performance characterization of megavoltage computed tomography imaging on a helical tomotherapy unit | 165 | 9.9 (9) |
| 9[[9](#_ENREF_9)] | 2005 | MP | Feasibility study of helical tomotherapy for total body or total marrow irradiation | 162 | 9.8 (10) |
| 10[[10](#_ENREF_10)] | 2004 | MP | Radiation characteristics of helical tomotherapy | 155 | 8.5 (13) |
| 11[[11](#_ENREF_11)] | 2007 | RAO | Adaptive biological image-guided IMRT with anatomic and functional imaging in pharyngo-laryngeal tumors: Impact on target volume delineation and dose distribution using helical tomotherapy | 133 | 9.1 (11) |
| 12[[12](#_ENREF_12)] | 2000 | PMB | Iterative approaches to dose optimization in tomotherapy | 132 | 5.9 (33) |
| 13[[13](#_ENREF_13)] | 2011 | RAO | Dosimetric comparison of left-sided whole breast irradiation with 3DCRT, forward-planned IMRT, inverse-planned IMRT, helical tomotherapy, and topotherapy | 128 | 11.9 (8) |
| 14[[14](#_ENREF_14)] | 2006 | RAO | Significant improvement in normal tissue sparing and target coverage for head and neck cancer by means of helical tomotherapy | 121 | 7.5 (20) |
| 15[[15](#_ENREF_15)] | 2002 | TCRT | Helical Tomotherapy: An Innovative Technology and Approach to Radiation Therapy | 117 | 5.9 (32) |
| 16[[16](#_ENREF_16)] | 2007 | IJROBP | Image-guided total marrow and total lymphatic irradiation using helical tomotherapy | 115 | 7.6 (19) |
| 17[[17](#_ENREF_17)] | 2009 | IJROBP | Image-guided total-marrow irradiation using helical tomotherapy in patients with multiple myeloma and acute leukemia undergoing hematopoietic cell transplantation | 112 | 8.4 (14) |
| 18[[18](#_ENREF_18)] | 2009 | RAO | Dosimetric comparison of four different external beam partial breast irradiation techniques: Three-dimensional conformal radiotherapy, intensity-modulated radiotherapy, helical tomotherapy, and proton beam therapy | 110 | 8.3 (16) |
| 19[[19](#_ENREF_19)] | 2008 | IJROBP | Intensity-modulated proton therapy versus helical tomotherapy in nasopharynx cancer: Planning comparison and NTCP evaluation | 109 | 8.0 (17) |
| 20[[20](#_ENREF_20)] | 2006 | IJROBP | Intensity-modulated radiation therapy (IMRT) dosimetry of the head and neck: A comparison of treatment plans using linear accelerator-based IMRT and helical tomotherapy | 109 | 6.9 (25) |
| 21[[21](#_ENREF_21)] | 2005 | MP | The helical tomotherapy thread effect | 103 | 6.1 (31) |
| 22[[22](#_ENREF_22)] | 2004 | IJROBP | The utility of megavoltage computed tomography images from a helical tomotherapy system for setup verification purposes | 101 | 5.8 (34) |
| 23[[23](#_ENREF_23)] | 2008 | SO | Helical tomotherapy - Experiences of the first 150 patients in Heidelberg | 100 | 7.0 (22) |
| 24[[24](#_ENREF_24)] | 2004 | PMB | Quality assurance of a helical tomotherapy machine | 95 | 5.3 (44) |
| 25[[25](#_ENREF_25)] | 2004 | BJR | Is tomotherapy the future of IMRT? | 92 | 5.1 (47) |
| 26[[26](#_ENREF_26)] | 2009 | IJROBP | Helical tomotherapy planning for left-sided breast cancer patients with positive lymph nodes: comparison to conventional multiport breast technique | 90 | 6.8 (26) |
| 27[[27](#_ENREF_27)] | 2005 | IJROBP | Comparing step-and-shoot IMRT with dynamic helical tomotherapy IMRT plans for head-and-neck cancer | 90 | 5.4 (43) |
| 28[[28](#_ENREF_28)] | 2000 | PMB | Megavoltage CT image reconstruction during tomotherapy treatments | 90 | 4.2 (65) |
| 29[[29](#_ENREF_29)] | 2006 | IJROBP | A technique for adaptive image-guided helical tomotherapy for lung cancer | 89 | 5.5 (39) |
| 30[[30](#_ENREF_30)] | 2011 | IJROBP | Assessing the role of volumetric modulated arc therapy (VMAT) relative to IMRT and helical tomotherapy in the management of localized, locally advanced, and post-operative prostate cancer | 85 | 7.9 (18) |
| 31[[31](#_ENREF_31)] | 2009 | JACMP | Comparing planning time, delivery time and plan quality for IMRT, RapidArc and tomotherapy | 84 | 6.8 (27) |
| 32[[32](#_ENREF_32)] | 2007 | RAO | A treatment planning study comparing whole breast radiation therapy against conformal, IMRT and tomotherapy for accelerated partial breast irradiation | 84 | 5.5 (37) |
| 33[[33](#_ENREF_33)] | 2001 | IJROBP | Forward or inversely planned segmental multileaf collimator IMRT and sequential tomotherapy to treat multiple dominant intraprostatic lesions of prostate cancer to 90 GY | 84 | 4.1 (68) |
| 34[[34](#_ENREF_34)] | 2001 | MP | A feasible method for clinical delivery verification and dose reconstruction in tomotherapy | 84 | 4.0 (72) |
| 35[[35](#_ENREF_35)] | 2009 | BJR | High-precision radiotherapy for craniospinal irradiation: evaluation of three-dimensional conformal radiotherapy, intensity-modulated radiation therapy and helical TomoTherapy | 82 | 6.6 (28) |
| 36[[36](#_ENREF_36)] | 2012 | RAO | Volumetric modulated arc therapy for nasopharyngeal carcinoma: A dosimetric comparison with TomoTherapy and step-and-shoot IMRT | 81 | 8.4 (15) |
| 37[[37](#_ENREF_37)] | 2007 | RAO | Significant reduction of acute toxicity following pelvic irradiation with Helical Tomotherapy in patients with localized prostate cancer | 80 | 5.4 (41) |
| 38[[38](#_ENREF_38)] | 2010 | MP | MapCHECK used for rotational IMRT measurements: Step-and-shoot, Tomotherapy, RapidArc | 75 | 6.3 (29) |
| 39[[39](#_ENREF_39)] | 2008 | RAO | Dosimetric comparisons of helical tomotherapy and step-and-shoot intensity-modulated radiotherapy in nasopharyngeal carcinoma | 75 | 5.5 (38) |
| 40[[40](#_ENREF_40)] | 2007 | IJROBP | Comparison of plan quality provided by intensity-modulated arc therapy and helical tomotherapy | 75 | 5.1 (46) |
| 41[[41](#_ENREF_41)] | 2007 | RAO | A dosimetric comparison of non-coplanar IMRT versus Helical Tomotherapy for nasal cavity and paranasal sinus cancer | 75 | 4.9 (49) |
| 42[[42](#_ENREF_42)] | 2011 | MD | Treatment and dosimetric advantages between VMAT, IMRT, and helical tomotherapy in prostate cancer | 74 | 6.9 (24) |
| 43[[43](#_ENREF_43)] | 2005 | IJROBP | Evaluation of image-guided helical tomotherapy for the retreatment of spinal metastases | 74 | 4.5 (55) |
| 44[[44](#_ENREF_44)] | 2010 | IJROBP | Tomotherapy and multifield intensity-modulated radiotherapy planning reduce cardiac doses in left-sided breast cancer patients with unfavorable cardiac anatomy | 73 | 6.3 (30) |
| 45[[45](#_ENREF_45)] | 2009 | IJROBP | A comprehensive assessment by tumor site of patient setup using daily MVCT imaging from more than 3,800 helical tomotherapy treatments | 71 | 5.4 (42) |
| 46[[46](#_ENREF_46)] | 2003 | MP | Clinical helical tomotherapy commissioning dosimetry | 70 | 3.8 (79) |
| 47[[47](#_ENREF_47)] | 2007 | IJROBP | A dosimetric comparison of accelerated partial breast irradiation techniques: Multicatheter interstitial brachytherapy, three-dimensional conformal radiotherapy, and supine versus prone helical tomotherapy | 68 | 4.6 (53) |
| 48[[48](#_ENREF_48)] | 2006 | TCRT | Clinical implementation of adaptive helical tomotherapy: A unique approach to image-guided intensity modulated radiotherapy | 68 | 4.4 (59) |
| 49[[49](#_ENREF_49)] | 2006 | MP | Automatic registration of megavoltage to kilovoltage CT images in helical tomotherapy: An evaluation of the setup verification process for the special case of a rigid head phantom | 66 | 4.3 (62) |
| 50[[50](#_ENREF_50)] | 2006 | AO | Feasibility report of image guided stereotactic body radiotherapy (IG-SBRT) with tomotherapy for early stage medically inoperable lung cancer using extreme hypofractionation | 66 | 4.2 (64) |
| 51[[51](#_ENREF_51)] | 2003 | AJCOCCT | Reduction in radiation dose to lung and other normal tissues using helical tomotherapy to treat lung cancer, in comparison to conventional field arrangements | 66 | 3.4 (90) |
| 52[[52](#_ENREF_52)] | 1999 | IJROBP | Comparison of intensity-modulated tomotherapy with stereotactically guided conformal radiotherapy for brain tumors | 66 | 2.9 (95) |
| 53[[53](#_ENREF_53)] | 2008 | IJROBP | Evidence of limited motion of the prostate by carefully emptying the rectum as assessed by daily MVCT image guidance with helical tomotherapy | 65 | 4.7 (52) |
| 54[[54](#_ENREF_54)] | 2013 | RO | Total Body Irradiation (TBI) using Helical Tomotherapy in children and young adults undergoing stem cell transplantation | 64 | 7.0 (21) |
| 55[[55](#_ENREF_55)] | 2011 | RO | Rotational IMRT techniques compared to fixed gantry IMRT and Tomotherapy: multi-institutional planning study for head-and-neck cases | 64 | 5.7 (35) |
| 56[[56](#_ENREF_56)] | 2009 | RAO | Helical tomotherapy for locoregional irradiation including the internal mammary chain in left-sided breast cancer: Dosimetric evaluation | 64 | 4.8 (51) |
| 57[[57](#_ENREF_57)] | 2005 | BJR | Helical tomotherapy for craniospinal radiation | 64 | 3.8 (81) |
| 58[[58](#_ENREF_58)] | 2010 | IJROBP | Treatment-related pneumonitis and acute esophagitis in non-small-cell lung cancer patients treated with chemotherapy and helical tomotherapy | 63 | 5.5 (40) |
| 59[[59](#_ENREF_59)] | 2011 | RAO | 'Plan of the day' adaptive radiotherapy for bladder cancer using helical tomotherapy | 62 | 5.6 (36) |
| 60[[60](#_ENREF_60)] | 2006 | IJROBP | Evaluation of two tomotherapy-based techniques for the delivery of whole-breast intensity-modulated radiation therapy | 61 | 3.8 (77) |
| 61[[61](#_ENREF_61)] | 2006 | IJROBP | Prostate contouring uncertainty in megavoltage computed tomography images acquired with a helical tomotherapy unit during image-guided radiation therapy | 60 | 3.8 (82) |
| 62[[62](#_ENREF_62)] | 2005 | RAO | Feasibility of cranio-spinal axis radiation with the Hi-Art tomotherapy system | 60 | 3.6 (85) |
| 63[[63](#_ENREF_63)] | 2010 | PMB | Two new DOSXYZnrc sources for 4D Monte Carlo simulations of continuously variable beam configurations, with applications to RapidArc, VMAT, TomoTherapy and CyberKnife | 59 | 5.0 (48) |
| 64[[64](#_ENREF_64)] | 2011 | IJROBP | SmartArc-based volumetric modulated arc therapy for oropharyngeal cancer: a dosimetric comparison with both intensity-modulated radiation therapy and helical tomotherapy | 57 | 5.3 (45) |
| 65[[65](#_ENREF_65)] | 2009 | RAO | Dosimetric assessment of static and helical TomoTherapy in the clinical implementation of breast cancer treatments | 57 | 4.5 (54) |
| 66[[66](#_ENREF_66)] | 2009 | IJROBP | Preoperative helical tomotherapy and megavoltage computed tomography for rectal cancer: impact on the irradiated volume of small bowel | 56 | 4.4 (57) |
| 67[[67](#_ENREF_67)] | 2006 | IJROBP | Dosimetric comparisons of helical tomotherapy treatment plans and step-and-shoot intensity-modulated radiosurgery treatment plans in intracranl stereotactic radiosurgery | 56 | 3.5 (89) |
| 68[[68](#_ENREF_68)] | 2001 | PMB | On the accuracy and effectiveness of dose reconstruction for tomotherapy | 56 | 2.7 (97) |
| 69[[69](#_ENREF_69)] | 1999 | PMB | Delivery verification in sequential and helical tomotherapy | 56 | 2.5 (100) |
| 70[[70](#_ENREF_70)] | 2009 | IJROBP | Phase I-II study of hypofractionated simultaneous integrated boost with tomotherapy for prostate cancer | 55 | 4.3 (63) |
| 71[[71](#_ENREF_71)] | 2008 | IJROBP | Phase II study of preoperative helical tomotherapy for rectal cancer | 55 | 3.9 (74) |
| 72[[72](#_ENREF_72)] | 2007 | SO | Simultaneous integrated boost (SIB) for nasopharynx cancer with helical tomotherapy - A planning study | 55 | 3.8 (83) |
| 73[[73](#_ENREF_73)] | 2007 | PMB | A motion phantom study on helical tomotherapy: the dosimetric impacts of delivery technique and motion | 55 | 3.6 (84) |
| 74[[74](#_ENREF_74)] | 2008 | RAO | Evaluating target coverage and normal tissue sparing in the adjuvant radiotherapy of malignant pleural mesothelioma: Helical tomotherapy compared with step-and-shoot IMRT | 54 | 3.8 (80) |
| 75[[75](#_ENREF_75)] | 2009 | N | Radiosurgery in the treatment of spinal metastases: tumor control, survival, and quality of life after helical tomotherapy | 53 | 4.3 (61) |
| 76[[76](#_ENREF_76)] | 2004 | PMB | Planning evaluation of radiotherapy for complex lung cancer cases using helical tomotherapy | 53 | 3.0 (94) |
| 77[[77](#_ENREF_77)] | 2019 | MP | A deep learning method for prediction of three-dimensional dose distribution of helical tomotherapy | 52 | 17.3 (3) |
| 78[[78](#_ENREF_78)] | 2010 | IJROBP | Standard and nonstandard craniospinal radiotherapy using helical TomoTherapy | 52 | 4.4 (58) |
| 79[[79](#_ENREF_79)] | 2008 | TCRT | Dose Escalated, Hypofractionated Radiotherapy Using Helical Tomotherapy for Inoperable Non-Small Cell Lung Cancer: Preliminary Results of a Risk-Stratified Phase I Dose Escalation Study | 52 | 3.9 (75) |
| 80[[80](#_ENREF_80)] | 2008 | RAO | Treatment planning comparison between conformal radiotherapy and helical tomotherapy in the case of locally advanced-stage NSCLC | 52 | 3.8 (78) |
| 81[[81](#_ENREF_81)] | 2007 | MD | Helical tomotherapy for radiotherapy in esophageal cancer: A preferred plan with better conformal target coverage and more homogeneous dose distribution | 52 | 3.5 (87) |
| 82[[82](#_ENREF_82)] | 2007 | IJROBP | A dosimetric comparison of electronic compensation, conventional intensity modulated radiotherapy, and tomotherapy in patients with early-stage carcinoma of the left breast | 52 | 3.5 (88) |
| 83[[83](#_ENREF_83)] | 2006 | SRO | Tomotherapy and other innovative IMRT delivery systems | 52 | 3.3 (91) |
| 84[[84](#_ENREF_84)] | 2008 | RAO | Dose painting with IMPT, helical tomotherapy and IMXT: A dosimetric comparison | 51 | 3.6 (86) |
| 85[[85](#_ENREF_85)] | 2003 | PMB | Optimization of helical tomotherapy treatment plans for prostate cancer | 51 | 2.7 (96) |
| 86[[86](#_ENREF_86)] | 2010 | IJROBP | Dynamic Jaws and Dynamic Couch in Helical Tomotherapy | 50 | 4.1 (66) |
| 87[[87](#_ENREF_87)] | 2010 | RAO | Simultaneous integrated boost in breast conserving treatment of breast cancer: A dosimetric comparison of helical tomotherapy and three-dimensional conformal radiotherapy | 50 | 4.1 (67) |
| 88[[88](#_ENREF_88)] | 2009 | IJROBP | Pediatric craniospinal axis irradiation with helical tomotherapy: patient outcome and lack of acute pulmonary toxicity | 50 | 4.0 (71) |
| 89[[89](#_ENREF_89)] | 2009 | MP | Monte Carlo evaluation of the convolution/superposition algorithm of Hi-Art (TM) tomotherapy in heterogeneous phantoms and clinical cases | 50 | 3.8 (76) |
| 90[[90](#_ENREF_90)] | 2012 | CO | Adaptive Radiotherapy Using Helical Tomotherapy for Head and Neck Cancer in Definitive and Postoperative Settings: Initial Results | 49 | 4.9 (50) |
| 91[[91](#_ENREF_91)] | 2011 | PMB | An automatic contour propagation method to follow parotid gland deformation during head-and-neck cancer tomotherapy | 49 | 4.4 (60) |
| 92[[92](#_ENREF_92)] | 2007 | IJROBP | A prospective evaluation of helical tomotherapy | 49 | 3.3 (92) |
| 93[[93](#_ENREF_93)] | 2003 | MP | Benchmarking beam alignment for a clinical helical tomotherapy device | 49 | 2.6 (99) |
| 94[[94](#_ENREF_94)] | 2015 | PM | Dosimetric comparison of left-sided whole-breast irradiation with 3DCRT, forward-planned IMRT, inverse-planned IMRT, helical tomotherapy, and volumetric arc therapy | 48 | 6.9 (23) |
| 95[[95](#_ENREF_95)] | 2011 | BMT | Clinical feasibility of TBI with helical tomotherapy | 48 | 4.4 (56) |
| 96[[96](#_ENREF_96)] | 2010 | SO | A Planning Comparison of Dynamic IMRT for Different Collimator Leaf Thicknesses with Helical Tomotherapy and RapidArc for Prostate and Head and Neck Tumors | 47 | 4.0 (70) |
| 97[[97](#_ENREF_97)] | 2010 | CO | Practical Aspects of Implementation of Helical Tomotherapy for Intensity-modulated and Image-guided Radiotherapy | 47 | 3.9 (73) |
| 98[[98](#_ENREF_98)] | 2011 | RAO | Helical tomotherapy targeting total bone marrow after total body irradiation for patients with relapsed acute leukemia undergoing an allogeneic stem cell transplant | 45 | 4.0 (69) |
| 99[[99](#_ENREF_99)] | 2008 | SO | Helical tomotherapy as a new treatment technique for whole abdominal irradiation | 45 | 3.2 (93) |
| 100[[100](#_ENREF_100)] | 2005 | RAO | Tomotherapy planning of small brain tumours | 45 | 2.6 (98) |

SRO = Seminars in radiation oncology; RAO = Radiotherapy and oncology; IJROBP = International journal of radiation oncology biology physics; PMB = Physics in medicine and biology; MP = Medical physics; TCRT = Technology in cancer research & treatment; SO = Strahlentherapie und onkologie; BJR = British journal of radiology; JACMP = Journal of applied clinical medical physics; MD = Medical dosimetry; AO = Acta oncologica; AJCOCCT = American journal of clinical oncology-cancer clinical trials; RO = Radiation oncology; N = Neurosurgery; CO = Clinical oncology; PM = Physica medica-european journal of medical physics; BMT = Bone marrow transplantation;

**Reference:**

1. Mackie TR, Balog J, Ruchala K, Shepard D, Aldridge S, Fitchard E, Reckwerdt P, Olivera G, McNutt T, Mehta M: **Tomotherapy**. *Seminars in radiation oncology* 1999, **9**(1):108-117.

2. Wolff D, Stieler F, Welzel G, Lorenz F, Abo-Madyan Y, Mai S, Herskind C, Polednik M, Steil V, Wenz F *et al*: **Volumetric modulated arc therapy (VMAT) vs. serial tomotherapy, step-and-shoot IMRT and 3D-conformal RT for treatment of prostate cancer**. *Radiotherapy and Oncology* 2009, **93**(2):226-233.

3. Gondi V, Tolakanahalli R, Mehta MP, Tewatia D, Rowley H, Kuo JS, Khuntia D, Tome WA: **Hippocampal-sparing whole-brain radiotherapy: a "how-to" technique using helical tomotherapy and linear accelerator-based intensity-modulated radiotherapy**. *International journal of radiation oncology, biology, physics* 2010, **78**(4):1244-1252.

4. Ruchala KJ, Olivera GH, Schloesser EA, Mackie TR: **Megavoltage CT on a tomotherapy system**. *Physics in medicine and biology* 1999, **44**(10):2597-2621.

5. Mackie TR: **History of tomotherapy**. *Physics in medicine and biology* 2006, **51**(13):R427-R453.

6. Rao M, Yang WS, Chen F, Sheng K, Ye JS, Mehta V, Shepard D, Cao DL: **Comparison of Elekta VMAT with helical tomotherapy and fixed field IMRT: Plan quality, delivery efficiency and accuracy**. *Medical physics* 2010, **37**(3):1350-1359.

7. Langen KM, Papanikolaou N, Balog J, Crilly R, Followill D, Goddu SM, Grant W, Olivera G, Ramsey CR, Shi CY: **QA for helical tomotherapy: Report of the AAPM Task Group 148**. *Medical physics* 2010, **37**(9):4817-4853.

8. Meeks SL, Harmon JF, Langen KM, Willoughby TR, Wagner TH, Kupelian PA: **Performance characterization of megavoltage computed tomography imaging on a helical tomotherapy unit**. *Medical physics* 2005, **32**(8):2673-2681.

9. Hui SK, Kapatoes J, Fowler J, Henderson D, Olivera G, Manon RR, Gerbi B, Mackie TR, Welsh JS: **Feasibility study of helical tomotherapy for total body or total marrow irradiation**. *Medical physics* 2005, **32**(10):3214-3224.

10. Jeraj R, Mackie TR, Balog J, Olivera G, Pearson D, Kapatoes J, Ruchala K, Reckwerdt P: **Radiation characteristics of helical tomotherapy**. *Medical physics* 2004, **31**(2):396-404.

11. Geets X, Tomsej M, Lee JA, Duprez T, Coche E, Cosnard G, Lonneux M, Gregoire V: **Adaptive biological image-guided IMRT with anatomic and functional imaging in pharyngo-laryngeal tumors: Impact on target volume delineation and dose distribution using helical tomotherapy**. *Radiotherapy and Oncology* 2007, **85**(1):105-115.

12. Shepard DM, Olivera GH, Reckwerdt PJ, Mackie TR: **Iterative approaches to dose optimization in tomotherapy**. *Physics in medicine and biology* 2000, **45**(1):69-90.

13. Schubert LK, Gondi V, Sengbusch E, Westerly DC, Soisson ET, Paliwal BR, Mackie TR, Mehta MP, Patel RR, Tome WA *et al*: **Dosimetric comparison of left-sided whole breast irradiation with 3DCRT, forward-planned IMRT, inverse-planned IMRT, helical tomotherapy, and topotherapy**. *Radiotherapy and Oncology* 2011, **100**(2):241-246.

14. Fiorino C, Dell'Oca I, Pierellia A, Broggi S, De Martin E, Di Muzio N, Longobardi B, Fazio F, Calandrino R: **Significant improvement in normal tissue sparing and target coverage for head and neck cancer by means of helical tomotherapy**. *Radiotherapy and Oncology* 2006, **78**(3):276-282.

15. Welsh JS, Patel RR, Ritter MA, Harari PM, Mackie TR, Mehta MP: **Helical Tomotherapy: An Innovative Technology and Approach to Radiation Therapy**. *Technology in cancer research & treatment* 2002, **1**(4):311-316.

16. Schultheiss TE, Wong J, Liu A, Olivera G, Somlo G: **Image-guided total marrow and total lymphatic irradiation using helical tomotherapy**. *International Journal of Radiation Oncology Biology Physics* 2007, **67**(4):1259-1267.

17. Wong JY, Rosenthal J, Liu A, Schultheiss T, Forman S, Somlo G: **Image-guided total-marrow irradiation using helical tomotherapy in patients with multiple myeloma and acute leukemia undergoing hematopoietic cell transplantation**. *International journal of radiation oncology, biology, physics* 2009, **73**(1):273-279.

18. Moon SH, Shin KH, Kim TH, Yoon M, Park S, Lee DH, Kim JW, Kim DW, Park SY, Cho KH: **Dosimetric comparison of four different external beam partial breast irradiation techniques: Three-dimensional conformal radiotherapy, intensity-modulated radiotherapy, helical tomotherapy, and proton beam therapy**. *Radiotherapy and Oncology* 2009, **90**(1):66-73.

19. Widesott L, Pierelli A, Fiorino C, Dell'Oca I, Broggi S, Catraneo GM, Di Muzio N, Fazio F, Calandrino R, Schwarz M: **Intensity-modulated proton therapy versus helical tomotherapy in nasopharynx cancer: Planning comparison and NTCP evaluation**. *International Journal of Radiation Oncology Biology Physics* 2008, **72**(2):589-596.

20. Sheng K, Molloy JA, Read PW: **Intensity-modulated radiation therapy (IMRT) dosimetry of the head and neck: A comparison of treatment plans using linear accelerator-based IMRT and helical tomotherapy**. *International Journal of Radiation Oncology Biology Physics* 2006, **65**(3):917-923.

21. Kissick MW, Fenwick J, James JA, Jerai R, Kapatoes JM, Keller H, Mackie TR, Olivera G, Soisson ET: **The helical tomotherapy thread effect**. *Medical physics* 2005, **32**(5):1414-1423.

22. Forrest LJ, Mackie TR, Ruchala K, Turek M, Kapatoes J, Jaradat H, Hui S, Balog J, Vail DM, Mehta MP: **The utility of megavoltage computed tomography images from a helical tomotherapy system for setup verification purposes**. *International Journal of Radiation Oncology Biology Physics* 2004, **60**(5):1639-1644.

23. Sterzing F, Schubert K, Sroka-Perez G, Kalz J, Debus J, Herfarth K: **Helical tomotherapy - Experiences of the first 150 patients in Heidelberg**. *Strahlentherapie Und Onkologie* 2008, **184**(1):8-14.

24. Fenwick JD, Tome WA, Jaradat HA, Hui SK, James JA, Balog JP, DeSouza CN, Lucas DB, Olivera GH, Mackie TR *et al*: **Quality assurance of a helical tomotherapy machine**. *Physics in medicine and biology* 2004, **49**(13):2933-2953.

25. Beavis AW: **Is tomotherapy the future of IMRT?** *British Journal of Radiology* 2004, **77**(916):285-295.

26. Goddu SM, Chaudhari S, Mamalui-Hunter M, Pechenaya OL, Pratt D, Mutic S, Zoberi I, Jeswani S, Powell SN, Low DA: **Helical tomotherapy planning for left-sided breast cancer patients with positive lymph nodes: comparison to conventional multiport breast technique**. *International journal of radiation oncology, biology, physics* 2009, **73**(4):1243-1251.

27. Van Vulpen M, Field C, Raaijmakers CPJ, Parliament MB, Terhaard CHJ, MacKenzie MA, Scrimger R, Lagendijk JJW, Fallone BG: **Comparing step-and-shoot IMRT with dynamic helical tomotherapy IMRT plans for head-and-neck cancer**. *International Journal of Radiation Oncology Biology Physics* 2005, **62**(5):1535-1539.

28. Ruchala KJ, Olivera GH, Kapatoes JM, Schloesser EA, Reckwerdt PJ, Mackie TR: **Megavoltage CT image reconstruction during tomotherapy treatments**. *Physics in medicine and biology* 2000, **45**(12):3545-3562.

29. Ramsey CR, Langen KM, Kupelian PA, Scaperoth DD, Meeks SL, Mahan SL, Seibert RM: **A technique for adaptive image-guided helical tomotherapy for lung cancer**. *International Journal of Radiation Oncology Biology Physics* 2006, **64**(4):1237-1244.

30. Davidson MT, Blake SJ, Batchelar DL, Cheung P, Mah K: **Assessing the role of volumetric modulated arc therapy (VMAT) relative to IMRT and helical tomotherapy in the management of localized, locally advanced, and post-operative prostate cancer**. *International journal of radiation oncology, biology, physics* 2011, **80**(5):1550-1558.

31. Oliver M, Ansbacher W, Beckham WA: **Comparing planning time, delivery time and plan quality for IMRT, RapidArc and tomotherapy**. *Journal of applied clinical medical physics* 2009, **10**(4):117-131.

32. Oliver M, Chen J, Wong E, Van Dyk J, Perera F: **A treatment planning study comparing whole breast radiation therapy against conformal, IMRT and tomotherapy for accelerated partial breast irradiation**. *Radiotherapy and Oncology* 2007, **82**(3):317-323.

33. Xia P, Pickett B, Vigneault E, Verhey LJ, Roach M: **Forward or inversely planned segmental multileaf collimator IMRT and sequential tomotherapy to treat multiple dominant intraprostatic lesions of prostate cancer to 90 GY**. *International Journal of Radiation Oncology Biology Physics* 2001, **51**(1):244-254.

34. Kapatoes JM, Olivera GH, Ruchala KJ, Smilowitz JB, Reckwerdt PJ, Mackie TR: **A feasible method for clinical delivery verification and dose reconstruction in tomotherapy**. *Medical physics* 2001, **28**(4):528-542.

35. Sharma DS, Gupta T, Jalali R, Master Z, Phurailatpam RD, Sarin R: **High-precision radiotherapy for craniospinal irradiation: evaluation of three-dimensional conformal radiotherapy, intensity-modulated radiation therapy and helical TomoTherapy**. *British Journal of Radiology* 2009, **82**(984):1000-1009.

36. Lu SH, Cheng JCH, Kuo SH, Lee JJS, Chen LH, Wu JK, Chen YL, Chen WY, Wen SY, Chong FC *et al*: **Volumetric modulated arc therapy for nasopharyngeal carcinoma: A dosimetric comparison with TomoTherapy and step-and-shoot IMRT**. *Radiotherapy and Oncology* 2012, **104**(3):324-330.

37. Cozzarini C, Fiorino C, Di Muzio N, Alongi F, Broggi S, Cattaneo M, Montorsi F, Rigatti P, Calandrino R, Fazio F: **Significant reduction of acute toxicity following pelvic irradiation with Helical Tomotherapy in patients with localized prostate cancer**. *Radiotherapy and Oncology* 2007, **84**(2):164-170.

38. Jursinic PA, Sharma R, Reuter J: **MapCHECK used for rotational IMRT measurements: Step-and-shoot, Tomotherapy, RapidArc**. *Medical physics* 2010, **37**(6):2837-2846.

39. Lee TF, Fang FM, Chao PJ, Su TJ, Wang LK, Leung SW: **Dosimetric comparisons of helical tomotherapy and step-and-shoot intensity-modulated radiotherapy in nasopharyngeal carcinoma**. *Radiotherapy and Oncology* 2008, **89**(1):89-96.

40. Cao D, Holmes TW, Afghan MKN, Shepard DM: **Comparison of plan quality provided by intensity-modulated arc therapy and helical tomotherapy**. *International Journal of Radiation Oncology Biology Physics* 2007, **69**(1):240-250.

41. Sheng K, Molloy JA, Larner JM, Read PW: **A dosimetric comparison of non-coplanar IMRT versus Helical Tomotherapy for nasal cavity and paranasal sinus cancer**. *Radiotherapy and Oncology* 2007, **82**(2):174-178.

42. Tsai CL, Wu JK, Chao HL, Tsai YC, Cheng JC: **Treatment and dosimetric advantages between VMAT, IMRT, and helical tomotherapy in prostate cancer**. *Medical dosimetry : official journal of the American Association of Medical Dosimetrists* 2011, **36**(3):264-271.

43. Mahan SL, Ramsey CR, Scaperoth DD, Chase DJ, Byrne TE: **Evaluation of image-guided helical tomotherapy for the retreatment of spinal metastases**. *International Journal of Radiation Oncology Biology Physics* 2005, **63**(5):1576-1583.

44. Coon AB, Dickler A, Kirk MC, Liao Y, Shah AP, Strauss JB, Chen S, Turian J, Griem KL: **Tomotherapy and multifield intensity-modulated radiotherapy planning reduce cardiac doses in left-sided breast cancer patients with unfavorable cardiac anatomy**. *International journal of radiation oncology, biology, physics* 2010, **78**(1):104-110.

45. Schubert LK, Westerly DC, Tome WA, Mehta MP, Soisson ET, Mackie TR, Ritter MA, Khuntia D, Harari PM, Paliwal BR: **A comprehensive assessment by tumor site of patient setup using daily MVCT imaging from more than 3,800 helical tomotherapy treatments**. *International journal of radiation oncology, biology, physics* 2009, **73**(4):1260-1269.

46. Balog J, Olivera G, Kapatoes J: **Clinical helical tomotherapy commissioning dosimetry**. *Medical physics* 2003, **30**(12):3097-3106.

47. Patel RR, Becker SJ, Das RK, Mackie TR: **A dosimetric comparison of accelerated partial breast irradiation techniques: Multicatheter interstitial brachytherapy, three-dimensional conformal radiotherapy, and supine versus prone helical tomotherapy**. *International Journal of Radiation Oncology Biology Physics* 2007, **68**(3):935-942.

48. Welsh JS, Lock M, Harari PM, Tome WA, Fowler J, Mackie TR, Ritter M, Kapatoes J, Forrest L, Chappell R *et al*: **Clinical implementation of adaptive helical tomotherapy: A unique approach to image-guided intensity modulated radiotherapy**. *Technology in cancer research & treatment* 2006, **5**(5):465-479.

49. Boswell S, Tome W, Jeraj R, Jaradat H, Mackie TR: **Automatic registration of megavoltage to kilovoltage CT images in helical tomotherapy: An evaluation of the setup verification process for the special case of a rigid head phantom**. *Medical physics* 2006, **33**(11):4395-4404.

50. Hodge W, Tome WA, Jaradat HA, Orton NP, Khuntia D, Traynor A, Weigel T, Mehta MP: **Feasibility report of image guided stereotactic body radiotherapy (IG-SBRT) with tomotherapy for early stage medically inoperable lung cancer using extreme hypofractionation**. *Acta oncologica* 2006, **45**(7):890-896.

51. Scrimger RA, Tome WA, Olivera GH, Reckwerdt PJ, Mehta MP, Fowler JF: **Reduction in radiation dose to lung and other normal tissues using helical tomotherapy to treat lung cancer, in comparison to conventional field arrangements**. *Am J Clin Oncol-Cancer Clin Trials* 2003, **26**(1):70-78.

52. Khoo VS, Oldham M, Adams EJ, Bedford JL, Webb S, Brada M: **Comparison of intensity-modulated tomotherapy with stereotactically guided conformal radiotherapy for brain tumors**. *International Journal of Radiation Oncology Biology Physics* 1999, **45**(2):415-425.

53. Fiorino C, Di Mum N, Broggi S, Cozzarini C, Maggiulli E, Alongi F, Valdagni R, Fazio F, Calandrino R: **Evidence of limited motion of the prostate by carefully emptying the rectum as assessed by daily MVCT image guidance with helical tomotherapy**. *International Journal of Radiation Oncology Biology Physics* 2008, **71**(2):611-617.

54. Gruen A, Ebell W, Wlodarczyk W, Neumann O, Kuehl JS, Stromberger C, Budach V, Marnitz S: **Total Body Irradiation (TBI) using Helical Tomotherapy in children and young adults undergoing stem cell transplantation**. *Radiation oncology* 2013, **8**.

55. Wiezorek T, Brachwitz T, Georg D, Blank E, Fotina I, Habl G, Kretschmer M, Lutters G, Salz H, Schubert K *et al*: **Rotational IMRT techniques compared to fixed gantry IMRT and Tomotherapy: multi-institutional planning study for head-and-neck cases**. *Radiation oncology* 2011, **6**.

56. Caudrelier JM, Morgan SC, Montgomery L, Lacelle M, Nyiri B, MacPherson M: **Helical tomotherapy for locoregional irradiation including the internal mammary chain in left-sided breast cancer: Dosimetric evaluation**. *Radiotherapy and Oncology* 2009, **90**(1):99-105.

57. Bauman G, Yartsev S, Coad T, Fisher B, Kron T: **Helical tomotherapy for craniospinal radiation**. *British Journal of Radiology* 2005, **78**(930):548-552.

58. Song CH, Pyo H, Moon SH, Kim TH, Kim DW, Cho KH: **Treatment-related pneumonitis and acute esophagitis in non-small-cell lung cancer patients treated with chemotherapy and helical tomotherapy**. *International journal of radiation oncology, biology, physics* 2010, **78**(3):651-658.

59. Murthy V, Master Z, Adurkar P, Mallick I, Mahantshetty U, Bakshi G, Tongaonkar H, Shrivastava S: **'Plan of the day' adaptive radiotherapy for bladder cancer using helical tomotherapy**. *Radiotherapy and Oncology* 2011, **99**(1):55-60.

60. Gonzalez VJ, Buchholz DJ, Langen KM, Olivera GH, Chauhan B, Meeks SL, Ruchala KJ, Haimerl J, Lu WG, Kupelian PA: **Evaluation of two tomotherapy-based techniques for the delivery of whole-breast intensity-modulated radiation therapy**. *International Journal of Radiation Oncology Biology Physics* 2006, **65**(1):284-290.

61. Song WY, Chiu B, Bauman GS, Lock M, Rodrigues G, Ash R, Lewis C, Fenster A, Battista JJ, Van Dyk J: **Prostate contouring uncertainty in megavoltage computed tomography images acquired with a helical tomotherapy unit during image-guided radiation therapy**. *International Journal of Radiation Oncology Biology Physics* 2006, **65**(2):595-607.

62. Penagaricano JA, Papanikolaou N, Yan YL, Youssef E, Ratanatharathorn V: **Feasibility of cranio-spinal axis radiation with the Hi-Art tomotherapy system**. *Radiotherapy and Oncology* 2005, **76**(1):72-78.

63. Lobo J, Popescu IA: **Two new DOSXYZnrc sources for 4D Monte Carlo simulations of continuously variable beam configurations, with applications to RapidArc, VMAT, TomoTherapy and CyberKnife**. *Physics in medicine and biology* 2010, **55**(16):4431-4443.

64. Clemente S, Wu B, Sanguineti G, Fusco V, Ricchetti F, Wong J, McNutt T: **SmartArc-based volumetric modulated arc therapy for oropharyngeal cancer: a dosimetric comparison with both intensity-modulated radiation therapy and helical tomotherapy**. *International journal of radiation oncology, biology, physics* 2011, **80**(4):1248-1255.

65. Reynders T, Tournel K, De Coninck P, Heymann S, Vinh-Hung V, Van Parijs H, Duchateau M, Linthout N, Gevaert T, Verellen D *et al*: **Dosimetric assessment of static and helical TomoTherapy in the clinical implementation of breast cancer treatments**. *Radiotherapy and Oncology* 2009, **93**(1):71-79.

66. Engels B, De Ridder M, Tournel K, Sermeus A, De Coninck P, Verellen D, Storme GA: **Preoperative helical tomotherapy and megavoltage computed tomography for rectal cancer: impact on the irradiated volume of small bowel**. *International journal of radiation oncology, biology, physics* 2009, **74**(5):1476-1480.

67. Pezner RD, Liu A, Han CH, Chen YJ, Schultheiss TE, Wong JYC: **Dosimetric comparison of helical tomotherapy treatment and step-and-shoot intensity-modulated radiotherapy of retroperitoneal sarcoma**. *Radiotherapy and Oncology* 2006, **81**(1):81-87.

68. Kapatoes JM, Olivera GH, Balog JP, Keller H, Reckwerdt PJ, Mackie TR: **On the accuracy and effectiveness of dose reconstruction for tomotherapy**. *Physics in medicine and biology* 2001, **46**(4):943-966.

69. Kapatoes JM, Olivera GH, Reckwerdt PJ, Fitchard EE, Schloesser EA, Mackie TR: **Delivery verification in sequential and helical tomotherapy**. *Physics in medicine and biology* 1999, **44**(7):1815-1841.

70. Di Muzio N, Fiorino C, Cozzarini C, Alongi F, Broggi S, Mangili P, Guazzoni G, Valdagni R, Calandrino R, Fazio F: **Phase I-II study of hypofractionated simultaneous integrated boost with tomotherapy for prostate cancer**. *International journal of radiation oncology, biology, physics* 2009, **74**(2):392-398.

71. De Ridder M, Tournel K, Van Nieuwenhove Y, Engels B, Hoorens A, Everaert H, De Beeck BO, Vinh-Hung V, De Greve J, Delvaux G *et al*: **Phase II study of preoperative helical tomotherapy for rectal cancer**. *International Journal of Radiation Oncology Biology Physics* 2008, **70**(3):728-734.

72. Fiorino C, Dell'Oca I, Pierelli A, Broggi S, Cattaneo GM, Chiara A, De Martin E, Di Muzio N, Fazio F, Calandrino R: **Simultaneous integrated boost (SIB) for nasopharynx cancer with helical tomotherapy - A planning study**. *Strahlentherapie Und Onkologie* 2007, **183**(9):497-505.

73. Kanagaki B, Read PW, Molloy JA, Larner JM, Sheng K: **A motion phantom study on helical tomotherapy: the dosimetric impacts of delivery technique and motion**. *Physics in medicine and biology* 2007, **52**(1):243-255.

74. Sterzing F, Sroka-Perez G, Schubert K, Munter MW, Thieke C, Huber P, Debus J, Herfarth KK: **Evaluating target coverage and normal tissue sparing in the adjuvant radiotherapy of malignant pleural mesothelioma: Helical tomotherapy compared with step-and-shoot IMRT**. *Radiotherapy and Oncology* 2008, **86**(2):251-257.

75. Sheehan JP, Shaffrey CI, Schlesinger D, Williams BJ, Arlet V, Larner J: **Radiosurgery in the treatment of spinal metastases: tumor control, survival, and quality of life after helical tomotherapy**. *Neurosurgery* 2009, **65**(6):1052-1061; discussion 1061-1052.

76. Kron T, Grigorov G, Yu E, Yartsev S, Chen JZ, Wong EG, Rodrigues G, Trenka K, Coad T, Bauman G *et al*: **Planning evaluation of radiotherapy for complex lung cancer cases using helical tomotherapy**. *Physics in medicine and biology* 2004, **49**(16):3675-3690.

77. Liu ZQ, Fan JW, Li MH, Yan H, Hu ZH, Huang P, Tian Y, Miao JJ, Dai JR: **A deep learning method for prediction of three-dimensional dose distribution of helical tomotherapy**. *Medical physics* 2019, **46**(5):1972-1983.

78. Parker W, Brodeur M, Roberge D, Freeman C: **Standard and nonstandard craniospinal radiotherapy using helical TomoTherapy**. *International journal of radiation oncology, biology, physics* 2010, **77**(3):926-931.

79. Adkison JB, Khuntia D, Bentzen SM, Cannon GM, Tome WA, Jaradat H, Walker W, Traynor AM, Weigel T, Mehta MP: **Dose Escalated, Hypofractionated Radiotherapy Using Helical Tomotherapy for Inoperable Non-Small Cell Lung Cancer: Preliminary Results of a Risk-Stratified Phase I Dose Escalation Study**. *Technology in cancer research & treatment* 2008, **7**(6):441-447.

80. Cattaneo GM, Dell'Oca I, Broggi S, Fiorino C, Perna L, Pasetti M, Sangalli G, di Muzio N, Fazio F, Calandrino R: **Treatment planning comparison between conformal radiotherapy and helical tomotherapy in the case of locally advanced-stage NSCLC**. *Radiotherapy and Oncology* 2008, **88**(3):310-318.

81. Chen YJ, Liu A, Han CH, Tsai PT, Schultheiss TE, Pezner RD, Vora N, Lim D, Shibata S, Kernstine KH *et al*: **Helical tomotherapy for radiotherapy in esophageal cancer: A preferred plan with better conformal target coverage and more homogeneous dose distribution**. *Medical Dosimetry* 2007, **32**(3):166-171.

82. Caudell JJ, De los Santos JF, Keene KS, Fiveash JB, Wang WQ, Carlisle JD, Popple R: **A dosimetric comparison of electronic compensation, conventional intensity modulated radiotherapy, and tomotherapy in patients with early-stage carcinoma of the left breast**. *International Journal of Radiation Oncology Biology Physics* 2007, **68**(5):1505-1511.

83. Fenwick JD, Tome WA, Soisson ET, Mehta MP, Mackie TR: **Tomotherapy and other innovative IMRT delivery systems**. *Seminars in radiation oncology* 2006, **16**(4):199-208.

84. Thorwarth D, Soukup M, Atber M: **Dose painting with IMPT, helical tomotherapy and IMXT: A dosimetric comparison**. *Radiotherapy and Oncology* 2008, **86**(1):30-34.

85. Grigorov G, Kron T, Wong E, Chen J, Sollazzo J, Rodrigues G: **Optimization of helical tomotherapy treatment plans for prostate cancer**. *Physics in medicine and biology* 2003, **48**(13):1933-1943.

86. Sterzing F, Uhl M, Hauswald H, Schubert K, Sroka-Perez G, Chen Y, Lu W, Mackie R, Debus J, Herfarth K *et al*: **Dynamic Jaws and Dynamic Couch in Helical Tomotherapy**. *International Journal of Radiation Oncology*Biology*Physics* 2010, **76**(4):1266-1273.

87. Hijal T, Fournier-Bidoz N, Castro-Pena P, Kirova YM, Zefkili S, Bollet MA, Dendale R, Campana F, Fourquet A: **Simultaneous integrated boost in breast conserving treatment of breast cancer: A dosimetric comparison of helical tomotherapy and three-dimensional conformal radiotherapy**. *Radiotherapy and Oncology* 2010, **94**(3):300-306.

88. Penagaricano J, Moros E, Corry P, Saylors R, Ratanatharathorn V: **Pediatric craniospinal axis irradiation with helical tomotherapy: patient outcome and lack of acute pulmonary toxicity**. *International journal of radiation oncology, biology, physics* 2009, **75**(4):1155-1161.

89. E S, F S, G O, S V: **Monte Carlo evaluation of the convolution/superposition algorithm of Hi-Art tomotherapy in heterogeneous phantoms and clinical cases**. *Medical physics* 2009, **36**(5):1566-1575.

90. Capelle L, Mackenzie M, Field C, Parliament M, Ghosh S, Scrimger R: **Adaptive Radiotherapy Using Helical Tomotherapy for Head and Neck Cancer in Definitive and Postoperative Settings: Initial Results**. *Clinical oncology* 2012, **24**(3):208-215.

91. Faggiano E, Fiorino C, Scalco E, Broggi S, Cattaneo M, Maggiulli E, Dell'Oca I, Di Muzio N, Calandrino R, Rizzo G: **An automatic contour propagation method to follow parotid gland deformation during head-and-neck cancer tomotherapy**. *Physics in medicine and biology* 2011, **56**(3):775-791.

92. Bauman G, Yartsev S, Rodrigues G, Lewis C, Venkatesan VM, Yu E, Hammond A, Perera F, Ash R, Dar AR *et al*: **A prospective evaluation of helical tomotherapy**. *International Journal of Radiation Oncology Biology Physics* 2007, **68**(2):632-641.

93. Balog J, Mackie TR, Pearson D, Hui S, Paliwal B, Jeraj R: **Benchmarking beam alignment for a clinical helical tomotherapy device**. *Medical physics* 2003, **30**(6):1118-1127.

94. Haciislamoglu E, Colak F, Canyilmaz E, Dirican B, Gurdalli S, Yilmaz AH, Yoney A, Bahat Z: **Dosimetric comparison of left-sided whole-breast irradiation with 3DCRT, forward-planned IMRT, inverse-planned IMRT, helical tomotherapy, and volumetric arc therapy**. *Phys Medica* 2015, **31**(4):360-367.

95. Penagaricano JA, Chao M, Van Rhee F, Moros EG, Corry PM, Ratanatharathorn V: **Clinical feasibility of TBI with helical tomotherapy**. *Bone Marrow Transplant* 2011, **46**(7):929-935.

96. Jacob V, Bayer W, Astner ST, Busch R, Kneschaurek P: **A Planning Comparison of Dynamic IMRT for Different Collimator Leaf Thicknesses with Helical Tomotherapy and RapidArc for Prostate and Head and Neck Tumors**. *Strahlentherapie Und Onkologie* 2010, **186**(9):502-510.

97. Burnet NG, Adams EJ, Fairfoul J, Tudor GSJ, Hoole ACF, Routsis DS, Dean JC, Kirby RD, Cowen M, Russell SG *et al*: **Practical Aspects of Implementation of Helical Tomotherapy for Intensity-modulated and Image-guided Radiotherapy**. *Clinical oncology* 2010, **22**(4):294-312.

98. Corvo R, Zeverino M, Vagge S, Agostinelli S, Barra S, Taccini G, Van Lint MT, Frassoni F, Bacigalupo A: **Helical tomotherapy targeting total bone marrow after total body irradiation for patients with relapsed acute leukemia undergoing an allogeneic stem cell transplant**. *Radiotherapy and Oncology* 2011, **98**(3):382-386.

99. Rochet N, Sterzing F, Jensen A, Dinkel J, Herfarth K, Schubert K, Eichbaum M, Schneeweiss A, Sohn C, Debus J *et al*: **Helical tomotherapy as a new treatment technique for whole abdominal irradiation**. *Strahlentherapie Und Onkologie* 2008, **184**(3):145-149.

100. Yaitsev S, Kron T, Cozzi L, Fogliata A, Bauman G: **Tomotherapy planning of small brain tumours**. *Radiotherapy and Oncology* 2005, **74**(1):49-52.
